# The analysis of psychosomatic disorders in medical students in the context of their exposure to traumatic events

**DOI:** 10.1192/j.eurpsy.2023.1017

**Published:** 2023-07-19

**Authors:** M. Kubiak, U. Szybowicz, M. Waszczak-Jeka, S. Jeka, P. Żuchowski, E. Mojs

**Affiliations:** 1 Department of Clinical Psychology, Poznań; 2 Clinical Medicine, Warsaw; 3 Collegium Medicum; 4 University Hospital no 2, Bydgoszcz, Poland

## Abstract

**Introduction:**

Stress is inextricably linked to mental well-being while stressful events remain a major contributor to many common psychosomatic disorders. Traumatic events are universal stressors. Only some individuals participating in stressful events do not develop full-blown post-traumatic stress disorder (PTSD) but many of them manifest psychosomatic symptoms with a strong psychological component

**Objectives:**

The current study compared the severity of somatization, anxiety, depression, and distress in medical university students who were exposed and in those who were not exposed to a traumatic event.

**Methods:**

Data were collected from 594 students of different faculties of the Poznan University of Medical Sciences in Poland. Participants were asked whether or not they had experienced any psychological trauma events and were asked to rate the intensity of psychosomatic symptoms they manifested. The data was collected using the Posttraumatic Diagnostic Scale (PDS) questionnaire and The Four-Dimensional Symptom Questionnaire (4 DSQ).

**Results:**

The study found that 78% of study participants experienced a traumatic event while 15% of them reported moderate and severe PTSD symptoms. 45% subjects reported moderate or high stress levels, 23% subjects reported symptoms of depression while 30% reported symptoms of anxiety. The analysis also demonstrated 26% of students participating in the study reported somatic symptoms.

In the subgroup of study participants with trauma history trauma sufferers, 36% subjects declared they experienced a one-time event, 23% subjects experienced trauma event twice while others experienced trauma ≥three times. The number of traumatic events was positively associated with the number of PTSD symptoms and severity of psychosomatic manifestations such as stress, depression, anxiety and somatization. In addition, the study analyzed whether traumatic events resulted from conscious and intentional harm by others. In this respect, 16% of subjects declared they participated in an event that was consciously and intentionally caused by others (e.g., battering or abuse). Students who experienced traumatic events related to intentionally harming another person were characterized by a greater severity of depression.

**Conclusions:**

Study indicates that experiencing traumatic events is associated with a greater severity of a range of psychosomatic symptoms.

**Disclosure of Interest:**

None Declared

